# Right ventricular outflow tract Doppler flow analysis and pulmonary arterial coupling by transthoracic echocardiography in sepsis: a retrospective exploratory study

**DOI:** 10.1186/s13054-022-04160-4

**Published:** 2022-10-03

**Authors:** Emma Maria Bowcock, Benjamin Gerhardy, Stephen Huang, Sam Orde

**Affiliations:** Intensive Care Medicine and Respiratory Medicine, Intensive Care Unit, Nepean Hospital, The University of Sydney, Derby Street, Penrith, Sydney, 2747 Australia

## Abstract

**Introduction:**

Right ventricular (RV) and pulmonary vascular dysfunction appear to be common in sepsis. RV performance is frequently assessed in isolation, yet its close relationship to afterload means combined analysis with right ventricular outflow tract (RVOT) Doppler and RV–pulmonary arterial (RV–PA) coupling may be more informative than standard assessment techniques. Data on feasibility and utility of these parameters in sepsis are lacking and were explored in this study.

**Methods:**

This is a retrospective study over a 3-year period of one-hundred and thirty-one patients admitted to ICU with sepsis who underwent transthoracic echocardiography (TTE) with RVOT pulsed wave Doppler. RVOT Doppler flow and RV–PA coupling was evaluated alongside standard measurements of RV systolic function and pulmonary pressures. RVOT Doppler analysis included assessment of pulmonary artery acceleration time (PAAT), velocity time integral and presence of notching. RV–PA coupling was assessed using tricuspid annular planar systolic excursion/pulmonary artery systolic pressure (TAPSE/PASP) ratio.

**Results:**

PAAT was measurable in 106 (81%) patients, and TAPSE/PASP was measurable in 77 (73%). Seventy-three (69%) patients had a PAAT of ≤ 100 ms suggesting raised pulmonary vascular resistance (PVR) is common. RVOT flow notching occurred in 15 (14%) of patients. TRV was unable to be assessed in 24 (23%) patients where measurement of PAAT was possible. RV dysfunction (RVD) was present in 28 (26%), 26 (25%) and 36 (34%) patients if subjective assessment, TAPSE < 17 mm and RV dilatation definitions were used, respectively. There was a trend towards shorter PAAT with increasing severity of RVD. RV–PA uncoupling defined as a TAPSE/PASP < 0.31 mm/mmHg was present in 15 (19%) patients. As RV dilatation increased the RV–PA coupling ratio decreased independent of LV systolic function, whereas TAPSE appeared to be more susceptible to changes in LV systolic function.

**Conclusion:**

Raised PVR and RV–PA uncoupling is seen in a significant proportion of patients with sepsis. Non-invasive assessment with TTE is feasible. The role of these parameters in assisting improved definitions of RVD, as well as their therapeutic and prognostic utility against standard parameters, deserves further investigation.

**Supplementary Information:**

The online version contains supplementary material available at 10.1186/s13054-022-04160-4.

## Key messages

- Increased pulmonary vascular resistance and RA-PA uncoupling is present in a significant subset of patients with sepsis, and measurement with TTE is feasible.
- Measurement of PAAT and RV-PA uncoupling could aid in improving definitions of right ventricular dysfunction.
- The therapeutic and prognostic role of these parameters in sepsis remains unknown, and further prospective studies are warranted.

**Supplementary Information:**

The online version contains supplementary material available at 10.1186/s13054-022-04160-4.

## Background

RVD is common in sepsis and seems to be associated with increased mortality [1, 2]. Patients can develop RVD as a consequence of left ventricular dysfunction; however, direct RV myocardial injury and increased RV afterload from increased pulmonary vascular resistance (PVR) are also important contributory factors [3–5]. The existing heterogeneity makes it difficult to direct targeted treatment strategies, and identification of more precise ‘RVD phenotypes’ is needed [6, 7]. RV performance is frequently assessed in isolation yet it is closely related to afterload; combined evaluation of the pulmonary circulation may be more beneficial at the bedside [8, 9]. Non-invasive assessment of RV–PA coupling using surrogates such as tricuspid annular planar systolic excursion/pulmonary artery systolic pressure (TAPSE/PASP) ratio has shown prognostic utility in recent studies [10, 11]. Data on feasibility, utility and prognostic significance in sepsis are lacking, however. Unfortunately, tricuspid regurgitant maximum velocity (TRV) is not always obtainable in the critically ill, making estimation of PASP difficult [12]. Evaluating the time the RV takes to achieve peak ejection, e.g. pulmonary artery acceleration time (PAAT), has utility in identifying those with raised PVR where TRV is not available [13, 14]. As we aim to improve definitions of RVD in sepsis, evaluation of RVOT Doppler flow alongside RV–PA coupling could provide rapid, non-invasive haemodynamic information to improve our understanding of RV performance. In addition, trending of these parameters could help identify patients with modifiable factors who may respond favourably to treatments that minimise PVR.

The objective of this exploratory study was to generate hypothesis for future prospective study in this area. We assessed RVOT Doppler waveform analysis and RV–PA coupling ratios and explored these parameters in relation to RV function, RV dilatation and pulmonary pressures.

## Methods

This is a single-centre, retrospective analysis of RVOT systolic flow profiles recorded by pulsed wave Doppler (PWD) in all patients (> 18 years) admitted with a primary diagnosis of sepsis who had a transthoracic echocardiogram (TTE) performed in ICU. Of 455 patients who were admitted to our unit with sepsis during July 2018–April 2021, 131 patients had a full transthoracic echo (TTE) study by cardiac sonographers that included PWD of the RVOT. Of those, 106 were sufficient for analysis. Those with congenital heart disease or known intracardiac shunts were excluded.

**Fig. 1:**
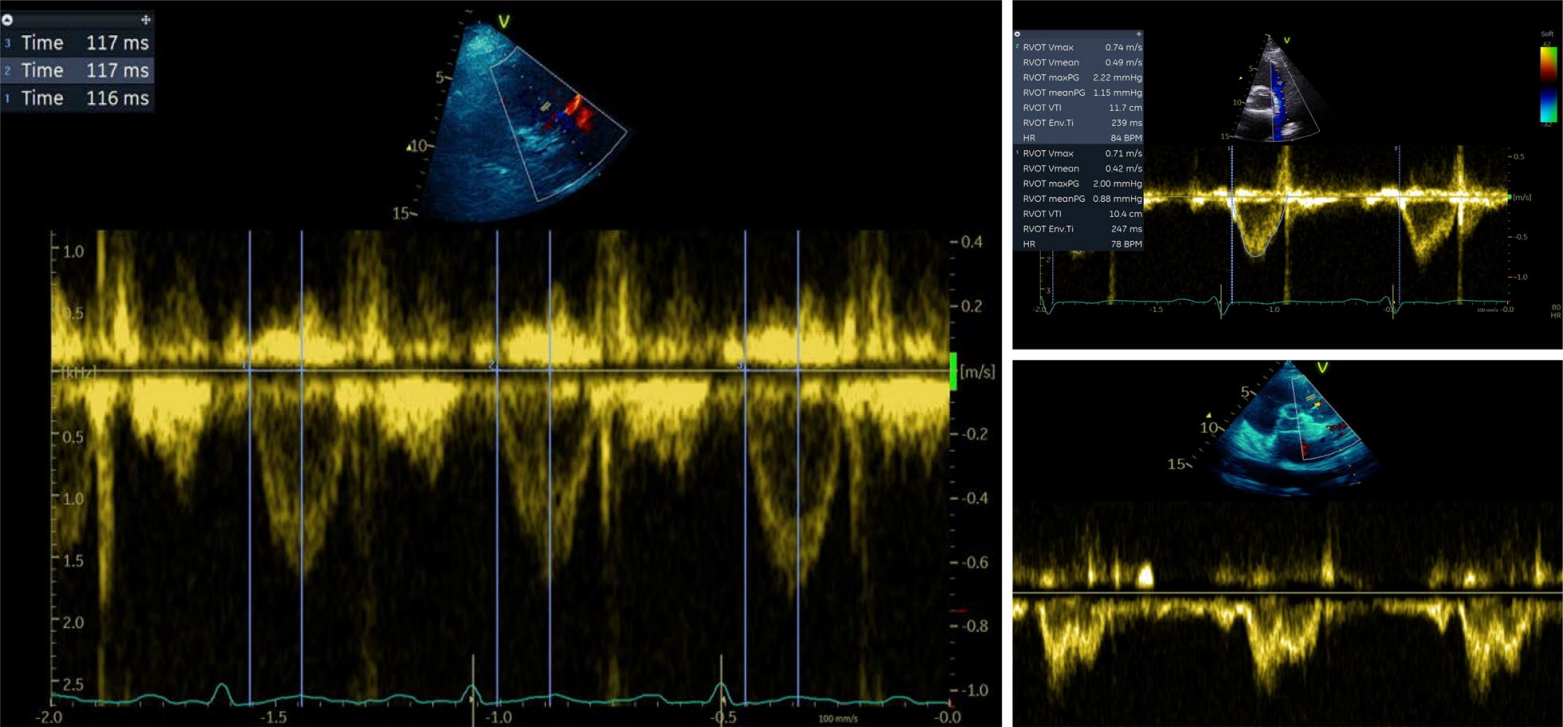
RVOT PWD profile. Left: measurement of PAAT. Top right: RVOT VTI and ET (RVOT Env Ti). Bottom right: “Notched” waveform showing Mid Systolic Notching (MSN)

Echo and clinical data were obtained from local intensive care echocardiography and clinical system databases, respectively. Measurement of PAAT, presence of 'notching’, RVOT ejection time (ET) and RVOT velocity time integral (VTI) were performed using an offline software analysis package by intensive care specialists with advanced echo qualification (EB, SO) (Fig. 1).

An average measurement of PAAT was taken from 3 (or 5 if in atrial fibrillation) consecutive RVOT Doppler profiles. PAAT corrected for heart rate (PAATc) was calculated using the formula PAAT × 75/HR [15]. Blinded interobserver variability for PAAT analysis was assessed by an echo trained specialist (BG) on a random 20% subgroup.

RVOT Doppler profiles were categorised as No notch (NN) and Notched (N) (Fig. 1). The notch position was defined by time-to-notch ratio, calculated as the time between the onset of the ejection and the notch to the ejection time. If this ratio was less than 0.66 (i.e. notch occurring within first 2/3rds of flow) and if subjective assessment agreed, the notch was characterised as midsystolic notching (MSN); others were characterised at late systolic notching (LSN) [16].

TAPSE/PASP was used as non-invasive measure of RV- PA coupling. A ratio of < 0.31 mm/mm Hg was used as the cut-off to define RV–PA uncoupling based on previously published data [17].

PASP was calculated by the Bernoulli equation using tricuspid regurgitant maximum velocity (TRV): (4xTRV^2^) + RAP (estimated to be 10 mmHg) [18]. Patients were divided into subgroups of severity: mild (35–49 mmHg), moderate (50–70 mmHg) and severe (> 70 mmHg). TRV was also used to detect PH as suggested in recent guidelines [13]. Values of > 2.8 m/s and > 3.4 m/s were used to represent possible and probable pulmonary hypertension (PH), respectively.

Three definitions of RVD were used: reduced systolic function by subjective assessment, decreased TAPSE of < 17 mm and RV dilatation. Subjectively reduced systolic function was further categorised as mild, moderate or severe. RV dilatation was assessed in apical 4 chamber views at end diastole and categorised as mild if the RV was greater than two thirds the size of the LV but smaller than the LV, moderate if the RV was the same size as the LV and severe if the RV was larger than the LV and apex forming.

Ethics approval was gained through local blue mountains health district research governance office. Consent was not required due to the retrospective nature of the study, and hence, a waiver of consent was sought.

### Statistical analysis

Normally distributed continuous variables were expressed as mean ± standard deviation (SD); skewed distributed continuous data were expressed as median and interquartile range (IQR); categorical variables were expressed as counts and percentages. For comparisons between continuous variables, a two-sided independent t test or Mann–Whitney U test was used for normally distributed and non-normally distributed variables, respectively. For comparison between categorical variables, contingency tables with X^2^ test, or Fisher’s exact test if less than 5, were used. Analysis of variance (ANOVA) testing was used to compare across more than 2 groups, and post hoc pairwise comparison was made using Tukey HSD. Normality was tested using Shapiro–Wilk test. Linear regression was used to analyse the relationship between TAPSE or TAPSE/PASP and RV size and was adjusted for LVEF (≥ 50% vs. < 50%). RV size was rated from 1 to 4 for normal size, mildly, moderately and severely dilated RV, respectively, and was assumed to be equally spaced and linearly related to RV end-diastolic area to LV end-diastolic area based on an earlier study [19]. Model validity (linearity, normality, independence and equal variance) was confirmed using standard methods. Interobserver variability for PAAT was assessed using Bland–Altman analysis for 20% of random individuals from the cohort. A *p* value < 0.05 was considered statistically significant. Statistical analyses were performed using Jamovi software 2022, version 2.3.

## Results

### Patient characteristics

The mean age was 66 ± 13 years with 57% male. 91% were alive at ICU discharge (*n* = 96), and 83% alive at hospital discharge (*n* = 88). Median length of ICU admission was 3.9 [4] days with no significant difference between ICU survivors and non-survivors. Median hospital length of stay was 14 (25) days in hospital survivors and 6.8 (13) days in nonsurvivors (*p* = 0.01). The median time from ICU admission to TTE was 2 (1.5) days in survivors and 2 (0.75) days in nonsurvivors, *p* = 0.24. Nine (8%) patients had sepsis of urinary tract origin. Thirty-three patients (31%) were mechanically ventilated, 85 (80%) patients received vasoactive support, and 13 (12%) received renal replacement therapy during their admission. Twelve (11%) patients were in atrial fibrillation at the time of TTE. APACHE3 score was significantly higher in ICU non-survivors. There was no significant difference in chronic comorbidities between ICU survivors and nonsurvivors, except for immunosuppression where ICU mortality was higher. Admission arterial partial pressure of oxygen/fraction of inspired oxygen ratio, partial pressure of carbon dioxide and lactate was not significantly different between ICU survivors and nonsurvivors. A table of results is provided in Additional file 1.

### Feasibility and interobserver variability

RVOT waveform analysis was possible in 106 (81%) of TTE studies with RVOT PWD traces. TRV was unavailable in 24 (23%) patients where RVOT Doppler flow measurement of PAAT was available. Time-to-notch ratio was able to be measured in all patients with notching of the RVOT waveform. Bland–Altman analysis showed acceptable agreement in PAAT measurement with a mean difference of 4 ms (upper and lower limits of agreement − 3.9 to 12 ms). RV–PA coupling using TAPSE/PASP ratio was measurable in 77 (73%).

### RVOT Doppler analysis

**Fig. 2:**
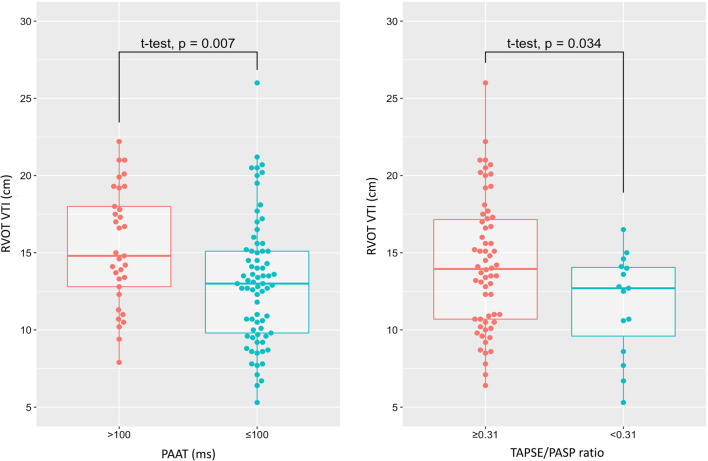
Linear model plots showing relationship of PAAT ≤ 100 ms (left) and RV–PA uncoupling (TAPSE/PASP ratio < 0.31 mm/mmHg) (right) with RVOT VTI. RVOT VTI is significantly lower in patients with a PAAT ≤ 100 ms, *p* = 0.007, and a TAPSE/PASP of < 0.31, *p* = 0.034

The mean PAAT was 91 ± 20 ms. Seventy-three patients (69%) had a PAAT of ≤ 100 ms, often used as a cut-off to suggest raised PVR. Those with a PAAT of ≤ 100 ms had significantly lower RVOT VTI (Fig. 2). Other values of RVOT Doppler analysis in relation to RV function by subjective assessment are shown in Table 1.

**Table 1:** Association of RVD by subjective assessment and RVOT analysis

RVD by subjective assessment severity groups	*N*	PAAT (ms)	RVOT VTI (cm)	RVOT ET (ms)
Normal	79	92 ± 21	15 ± 4	277 ± 49
Mild	16	89 ± 18	13 ± 3	264 ± 57
Moderate	9	86 ± 13	10 ± 2	280 ± 53
Severe	3	63 ± 11	6 ± 1	188 ± 91
*p* value		0.08	< 0.001	0.4

Mean ± standard deviation. RV systolic function groups: 1 = normal, 2 = mild, 3 = moderate, 4 = severe dysfunction

No significant correlation was seen between PAAT and admission Pa02, PaC02 and pH (r 0.2, *r* − 0.07, r 0.17, respectively). Differences in RVOT Doppler analysis by P/F ratio severity categories are shown in Additional file 2.

A notched RVOT Doppler profile was detectable in 15 patients (14%). PAAT was significantly shorter in those with notching (76 ± 14 vs. 93 ± 20 ms, *p* = 0.002). Fourteen (93%) of those with notched profiles had a PAAT of ≤ 100 ms. Eleven patients (73%) had midsystolic notching (MSN).

There was a trend towards increased incidence of notching of the RVOT profile in those with RVD by subjective assessment (OR 2.9 [CI 0.9–9], *p* = 0.06) but not in those with RV dilatation (OR 1.4 [CI 0.4–4.2], *p* = 0.57). Those with RVD defined by TAPSE < 17 mm were more likely to have notching (OR 3.5 [CI 1.1–11.3], *p* = 0.05). In those with RV–PA uncoupling (defined by TAPSE/PASP ratio of < 0.31 mm/mmHg), the presence of notching was significantly more likely (OR 7.6 [CI 1.9–30], *p* = 0.005).

#### Right ventricular dysfunction

RVD was present in 28 (26%), 26 (25%) and 36 (34%) patients if subjective, TAPSE < 17 mm and RV dilatation was used, respectively. As expected, RVOT VTI was significantly lower as RVD severity increased (*p* < 0.001) (Table 1). Post hoc pairwise comparison showed this was statistically significant across normal to moderate (*p* = 0.006), normal to severe (*p* = 0.002) and mild to severe (*p* = 0.04) severity subgroups. TAPSE was significantly correlated with RVOT VTI (*r* = 0.44, *p* < 0.001).

There was a trend towards shorter PAAT with increasing RVD severity by subjective assessment that was not statistically significant (*p* = 0.08) (Table 1). In those with RVD defined by TAPSE < 17 mm, there was no difference in PAAT (87 ± 20 vs. 93 ± 21 ms, *p* = 0.24) and there was poor correlation between TAPSE and PAAT (r 0.16, *p* = 0.10). There was no difference in PAAT in those with RV dilatation (92 ± 21 vs. 90 ± 19 ms, *p* = 0.75). Differences were not significant when PAAT was corrected for HR (PAATc).

There was no difference in admission P/F ratio in those with RVD defined by either subjective, dilatation, TAPSE or RV–PA uncoupling (Additional file 2). However, RVOT VTI and RVOT ET were significantly lower in those with worsening hypoxia defined by P/F ratio categories (Additional file 2). There was no difference in RVD by subjective assessment between patients who did and did not receive mechanical ventilation (*p* = 0.73). There was a trend towards increased RV dilatation in those who received mechanical ventilation that was not statistically significant (*p* = 0.06). There was no difference in TAPSE (20 ± 6 vs. 19 ± 4, *p* = 0.40) or RV–PA uncoupling in those that received mechanical ventilation. Further RV function and RVOT Doppler data in those who received mechanical ventilation during ICU admission can be found in Additional file 3.

There was a lower admission pH in those with RVD defined by TAPSE < 17 mm (7.32 ± 0.07 vs. 7.36 ± 0.09, *p* = 0.04, respectively) and a higher C02 in those with TAPSE < 17 mm [39.5 mmHg (11.8) vs. 37 mmHg (18.8), respectively, *p* = 0.04]. Further data are provided in Additional file 4.

### RV–PA coupling

RV–PA coupling was measurable in 77 (73%) of patients. RV–PA uncoupling, defined by a TAPSE/PASP ratio of < 0.31 mm/mmHg, was present in 15 patients (19%). PAAT was significantly lower in those with RV–PA uncoupling: 74 (14) vs. 92(20) ms, *p* = 0.009. PAAT was significantly more likely to be ≤ 100 ms in those with RV–PA uncoupling (OR 7.1 [CI 0.9–58], *p* = 0.05) as compared to other definitions of RVD. The correlation between TAPSE/PASP and PAAT was moderate but significant (r 0.4, *p* < 0.001).

Comparison of RV–PA coupling ratios to traditional assessments of RVD revealed significantly lower RV–PA coupling ratios in those with RVD by subjective assessment and RV dilatation: TAPSE/PASP ratio (0.29 (0.16) vs. 0.53(0.21), *p* < 0.001) and TAPSE/PASP ratio (0.34(0.23) vs. 0.52(0.22), *p* < 0.001), respectively. In patients with RV–PA uncoupling, the odds ratios for RV dilatation and RVD by subjective assessment were (9.8[CI 2.5–39], *p* < 0.001) and (14.3[CI 3.8–54], *p* < 0.001), respectively. Those with RV–PA uncoupling had lower RVOT VTI values as shown in Fig. 2. Other echo parameters in those with RV–PA uncoupling are shown in Table 2.

**Table 2:** Echocardiographic findings in those with RV–PA coupling (TAPSE/PASP ≥ 0.31 mmHg) and uncoupling (TAPSE/PASP < 0.31 mmHg)

Echocardiographic parameters	TAPSE/PASP < 0.31 mmHg (*n* = 15)	TAPSE/PASP ≥ 0.31 mmHg (*n* = 62)	*p* value
PAAT (ms)	79 (19)	92 (25)	0.004
PAATc (ms)	63 (14)	80 (46)	0.004
RVOT ET (ms)	263 ± 73	276 ± 80	0.43
RVOT VTI (cm)	12 ± 3	14 ± 4	0.03
LV ejection fraction (%)	55 (15)	55 (15)	0.73
E/e′	12 (13)	10 (6)	0.07
E/A	1.06 (0.9)	1.02 (0.6)	0.43
Lateral e′ (cm/s)	10 (3)	9 (5)	0.38
Medial e′ (cm/s)	6 (2)	7 (3)	0.07

Data presented as mean ± standard deviation or median (interquartile range)

**Fig. 3:**
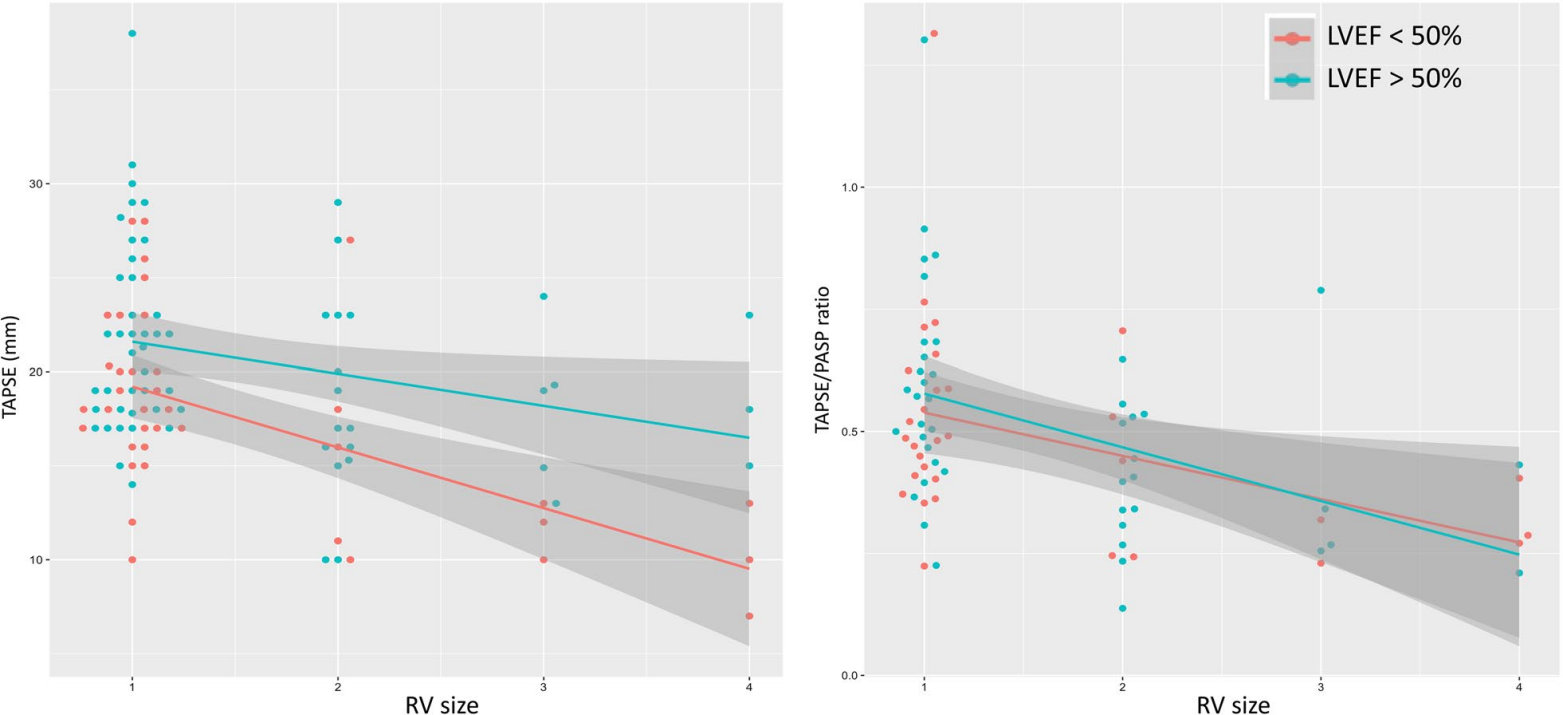
Linear regression plots showing a downtrend in TAPSE and TAPSE/PASP as RV dilatation severity increases. When dichotomised into those with normal (LVEF > 50%) and reduced LV systolic function (LVEF < 50%), there is an appreciable difference in TAPSE showing lower TAPSE values in those reduced LV systolic function (left). In contrast, LV systolic dysfunction does not appear to alter the TAPSE/PASP ratio relationship with RV size (right). RV size: 1 = normal, 2 = mild dilatation, 3 = moderate dilatation, 4 = severe dilatation

Figure 3 shows the relationships between TAPSE or TAPSE/PASP and RV size. The linear regression results demonstrated a negative association of TAPSE with RV size, with TAPSE reduced by 2.4 [− 3.5, − 1.3] mm on average for every increase step increase in RV size when LVEF was kept constant. On the other hand, patients with LVEF ≥ 50% exhibited a higher TAPSE, on average 3.2 [1.2, 5.2] mm higher than those with LVEF < 50%. TAPSE/PASP also displayed a similar negative relationship with RV size, with the ratio decreasing by 0.10 [− 0.15, − 0.04] for every step increase in RV size. However, TAPSE/PASP was unaffected by LVEF and the difference between LVEF ≥ 50% and < 50% was 0.03 [− 0.07, 0.12] units.

### Pulmonary pressures

Pulmonary hypertension (PH), defined as PASP ≥ 35 mmHg, was present in 56 (68%). A TRV of > 2.8 m/s was present in 37 (45%) and a TRV of > 3.4 m/s was present in 10 (12%). A moderate but significant inverse correlation was found between PAAT or PAATc and 4xTRV^2^ (*r* 0.31, *p* = 0.002 and *r* 0.34, *p* < 0.001, respectively). PASP was significantly higher in those with RVOT Doppler notching (51 ± 15 mmHg vs. 40 ± 11 mmHg, *p* = 0.003).

Severity subgroups of PH were as follows: mild (35–49 mmHg) in 37 (35%), moderate (50–70 mmHg) in 15 (14%) and severe (> 70 mmHg) in 3 (2.8%). Analysis of variance between RVD subjective severity and RV dilatation subgroups revealed no significant difference in PASP values (*p* = 0.4 and *p* = 0.12, respectively). There was no significant difference in PASP in those with RVD by TAPSE < 17 mms (*p* = 0.7).

As expected, there was a significantly shorter PAAT with increasing severity of PH by subgroup analysis of variance (p 0.03). Post hoc pairwise comparison revealed a significant difference between those with normal to mild (*p* = 0.003) and normal to severe PH (*p* = 0.05).

### LV systolic function

Left ventricular systolic function was decreased in 29 (27%) patients defined by an ejection fraction (EF) of < 50%. 11 (10%) had combined reduced LVEF and RVD by subjective assessment. PAAT was not significantly different in those with normal or reduced EF (86 ± 17 vs. 92 ± 21 ms, *p* = 0.2). TAPSE was significantly lower in those with reduced LV systolic function (18 ± 5 vs. 21 ± 5 mm, *p* = 0.005). There was no significant difference in RV–PA uncoupling in those with reduced LV systolic function [TAPSE/PASP 0.46 (0.23) vs. 0.5 (0.28), *p* = 0.69].

### Mortality

There was a trend towards increased ICU mortality in those with PAAT ≤ 100 ms that was not statistically significant (OR 4.5[CI 0.5–37], *p* = 0.17). Hospital mortality was significantly higher in those with PAAT ≤ 100 ms (OR 9.7[CI 1.2–76], *p* = 0.01). In those with TAPSE/PASP < 0.31 mm/mmHg, there was a trend towards increased ICU and hospital mortality that was not statistically significant (OR 4.9[CI 0.9–27], *p* = 0.08 and OR 3.4[CI 0.9–12.4] *p* = 0.12, respectively).

ICU and hospital mortality odds ratios were not significant for TAPSE < 17 mm, subjective RVD or RV dilatation: (OR 1.5[CI 0.3–6.4], *p* = 0.7 and OR 1.7[CI 0.6–5.2], *p* = 0.4; OR 1.2[CI 0.3–5], *p* = 0.7 and OR 2[CI 0.7–5.9], *p* = 0.2 and OR 1.7[CI 0.6–5.2],* p* = 0.4; OR 1.3[CI 0.3–5], *p* = 0.7 and OR 1.7[CI 0.6–4.8], *p* = 0.3, respectively).

## Discussion

In this single-centre, exploratory study we demonstrated that analysis of the RVOT Doppler waveform and RV–PA coupling is feasible, and our findings generate interesting questions with regards to defining RVD in sepsis.

Mechanisms of RVD in sepsis are complex, incompletely understood and occur because of concomitant LV systolic dysfunction, direct RV myocardial injury and increased RV afterload [2, 5, 20, 21]. Identifying echocardiographic parameters to detect early RVD and monitor the response to interventions is a key goal. There is a knowledge gap around non-invasive echo surrogates of pulmonary vascular dysfunction and its link to RV function in sepsis, and this study adds important observation in this area.

Vieillard Baron et al. showed that in patients with septic shock TAPSE alone was not able to discriminate between those with and without RV failure [22]. In a study evaluating patterns of RVD in ninety patients with COVID-19 ARDS [23], TAPSE was often normal in those with significant RVD. In comparison, assessment of RV function using fractional area change (FAC) performed better in detecting RVD, and interestingly, FAC/right ventricular systolic pressure correlated significantly with echo measurement of PVR and RV size. The authors suggested RV–PA coupling parameters may be more informative than standard measures alone [23]. There has been increasing interest in the use of TAPSE/PASP in sepsis. Zhang et al. found that a TAPSE/PASP ≤ 0.5 mm/mmHg was independently associated with increased ICU and 1 year mortality in mechanically ventilated patients with sepsis [24].

Invasive measures of RV–PA coupling from pressure–volume loop-derived end-systolic elastance (Ees)/end-systolic to arterial elastances (Ea) using right heart catheterisation (RHC) are considered gold standard but are impractical at the bedside. Animal studies have shown that with increasing severity of septic shock the initial preservation of RV–PA coupling is lost and progression to uncoupling occurs [25]. TAPSE provides an estimate of RV contractility and PASP an estimate of RV afterload, and thus, TAPSE/PASP has been proposed as a non-invasive surrogate of RV–PA coupling [17]. Tello et al. have shown that a TAPSE/PASP ratio of < 0.31 mm/mmHg outperformed other non-invasive ratios and independently correlated with invasive Ees/Ea uncoupling [17]. Lower tricuspid annular systolic velocity/right ventricular systolic pressure (TASV/RVSP) ratios were associated with increased mortality in a large retrospective data set of 4259 patients in cardiac intensive care that included patients with sepsis [10]. Overall data are emerging that metrics of RV–PA coupling may have added utility in those with RVD.

**Fig. 4:**
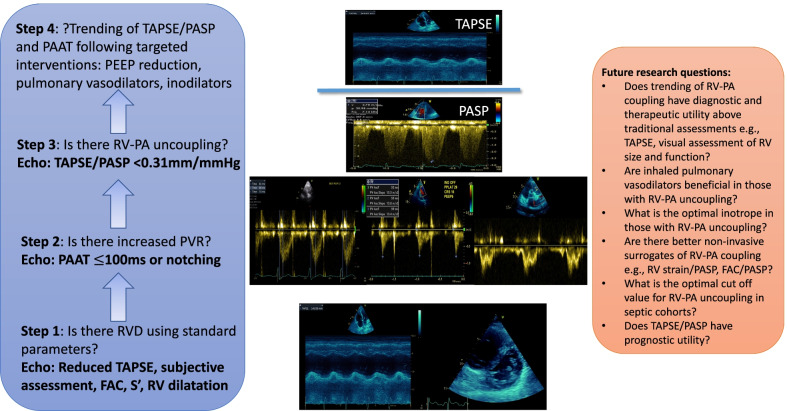
Proposed incremental role of PAAT and TAPSE/PASP in identifying ‘RV dysfunction phenotypes’, i.e. those with concomitant high PVR and/or RV–PA uncoupling. PVR, pulmonary vascular resistance; FAC, fractional area change; S′ = Tissue Doppler RV S′

In our study, we found that those with RV- PA uncoupling (defined as TAPSE/PASP $<$ 0.31 mm/mmHg) had significantly shorter PAAT and increased incidence of RVOT flow notching signalling that raised PVR is likely to be implicated in RV–PA uncoupling in sepsis. There was a trend towards lower RV–PA coupling ratios with increasing severity of RV dilatation. This finding suggests worsening RVD is potentially detectable by tracking values of TAPSE/PASP in a subset of patients. Perhaps it is in the subset of patients with RVD and RV–PA uncoupling that therapies aimed at reducing PVR (pulmonary vasodilators) or minimising PVR (earlier use of vasopressin with pulmonary sparing effects) may be of increased clinical benefit. The finding of the apparent independence of TAPSE/PASP and not TAPSE to LV systolic function is of interest (Fig. 3). It signals a potential benefit of this parameter over TAPSE alone in trending RV–PA interactions independent of LV systolic function. This could be particularly useful in those with biventricular dysfunction, e.g. in those with septic cardiomyopathy or pre-existing LV systolic dysfunction. Moving from isolated RV assessment to evaluation that incorporates the inextricable linkage to the pulmonary vasculature could have additive utility in trending RV performance above standard parameters (Fig. 4). The findings in this current study generate more questions than answers and ongoing research questions are proposed in Fig. 4. Prospective studies evaluating these knowledge gaps are needed if we are to improve definitions of RVD and advance towards having non-invasive bedside parameters to trend the impact of individualised targeted treatment strategies.

There are no prospective studies in critical care validating semi-invasive measures of RV–PA coupling using hybrid techniques evaluated in the non-critically ill such as TAPSE/invasive PVR [26], FAC/invasive mean pulmonary artery pressure or RV ejection fraction/ invasive PVR [27]. It would be of great interest to assess the feasibility and utility of these hybrid coupling ratios as well as their correlation to more practical non-invasive measurements such TAPSE/PASP, TAPSE/PAAT or FAC/PASP in future prospective study.

There exist conflicting findings regarding the prognostic impact of RVD on short term survival in sepsis [1, 2, 28, 29]. The heterogeneity of populations studied, poorly defined RVD categories, timing of echocardiography to include single snapshots of RVD are likely contributors to the divergent findings [1]. The low incidence of mortality and small numbers limits meaningful conclusions of the prognostic significance of RVD in this study. The trend towards increased short term mortality in those with PAAT ≤ 100 ms, and TAPSE/PASP < 0.31 mm/mmHg is of interest, however. Overall, a standardised assessment for RV studies as described by Huang et al. in the PRICES expert statement, perhaps with inclusion of RVOT and RV–PA coupling parameters, could help mitigate some of the issues outlined in future prospective studies [30].

Various cut-off values of PAAT ranging from 90 to 105 ms to detect raised PVR and/or PH have been described in the non-critically ill [31]. Tossavavinen et al. showed a PAAT less than 90 ms had superior accuracy (83%) to other non-invasive measures in identifying patients with raised PVR of > 3 WU when compared to RHC [14]. A PAAT $\le$ 100 ms was present in a high proportion (69%) of patients suggesting raised PVR is common in sepsis. The finding of increased RV–PA uncoupling but not RV dilatation or reduced TAPSE in those with PAAT ≤ 100 ms is not altogether unsurprising but is of interest. In keeping with data published by Bleakley et al. [23], it signals that there is an RV adaptation to raised PVR in sepsis that is perhaps better detected by measures of RV–PA coupling over traditional measures of RVD to include TAPSE. Although challenging, validation studies using TTE measured PAAT, PASP, TAPSE/PASP and RV function against invasively measured PVR, PASP and RV ejection fraction with fast response PA catheters could be useful to further inform this relationship in the critically ill.

TRV to estimate pulmonary pressures was unavailable in 24 (23%) patients where RVOT Doppler flow measurement of PAAT was available. This is an important finding as we know that a significant proportion of patients with pulmonary vascular dysfunction will be missed if pulmonary pressures derived from TRV are relied upon in isolation [15, 32]. It emphasises that PAAT should be measured in all critically ill patients to avoid missing those with significant pulmonary vascular dysfunction especially where TRV is either missing or inadequate [15, 32]. Yared et al. [33] found a strong inverse correlation (*r* − 0.95) between TTE measured PAAT and 4xTRV^2^ in the non-critically ill. In contrast, a moderate inverse correlation was found in our study. These conflicting results may be accounted for by the vastly different patient populations studied. Critically ill patients have varying degrees of RVD, tricuspid regurgitation severity and often inadequate spectral Doppler traces, all of which may affect values of TRV for calculation of PASP.

In the presence of increased PVR, reflected waves propagate more rapidly, with less attenuation, arriving at the RVOT during systole and causing systolic notching (Fig. 1). Systolic notching can be categorised midsystolic (MSN) or late systolic notching (LSN). The presence of MSN is more likely to represent increased pulmonary vascular resistance and poor vascular compliance. Those with a MSN pattern have the most severe pulmonary vascular disease and worst RV function in some studies [16]. Takathama et al. found signal notching, shortening of the midsystolic deceleration time and diminution of the late systolic flow velocity confer a higher mortality risk in those with PH [34]. In our study, 14% of patients had evidence of RVOT flow notching, and this was mostly MSN, suggesting significantly raised PVR. Those with RV–PA uncoupling were more likely to have notching; however, unexpectedly those with RV dilatation did not have increased incidence of notching. Further prospective study of RVOT notching patterns and PAAT alongside invasively measured PVR in those with preserved RV–PA coupling versus RV–PA uncoupling would be of interest and could improve our understanding of RVD phenotypes in sepsis.

## Limitations

There are several limitations in this present study that are inherent to its retrospective, single-centre design. As such, conclusions drawn can be hypothesis generating only. Small numbers and lack of multivariate analysis means conclusion about survival and echo parameters is limited.

The small number of patients limited the utility of multivariate analysis and exploration of cut-off values for RV–PA coupling and PAAT by receiver operating curve analysis. Only those who had a TTE were included introducing possible selection bias. The type and dose of haemodynamic support and loading conditions at the time of TTE were not known. Given many of the echo parameters assessed are load dependent, interpretation and generalisability of the findings are limited. The ventilator settings and gas exchange, in particular driving pressure, plateau pressure, PEEP, PaC0_2_ and Pa02/Fi02 ratio, were not known at the time of echo and may have influenced values obtained. The evaluation of RVD by subjective assessment in a single-centre introduces bias. However, subjective assessment of size and function are commonly practiced at the bedside and therefore it is a pragmatic parameter to evaluate. In addition, the finding of significantly lower RVOT VTI as subjective RVD severity increased offers some reassurance that qualitative assessment correlated with semi-quantitative measurement techniques. The relatively small number of patients with moderate to severe RVD limits generalisability of the findings. Importantly, our data do not allow RVD induced or worsened by respiratory failure to be distinguished from direct effects of sepsis or pre-existing RVD.

It was not known if the RVOT PWD was taken during expiration or inspiration and respiratory variation can significantly alter values obtained. Though it is suggested that taking an average of 3- 4 consecutive cycles, as was done in this study, is a reasonable method to mitigate the effects of respiratory variation [12]. Chronic co-morbidities of patients were unknown including pre-existing PH, RVD, diastolic dysfunction and valvular disease, and these are important factors that are likely to impact measurements. The lack of echocardiographic parameters such as RV end diastolic/LV end diastolic area ratio, eccentricity index, RV strain, RV fractional area change, RV systolic velocity (s’), RVOT area, TR severity, IVC size and collapsibility as well as hepatic and portal venous flow patterns means important measures of RV performance are missing. In addition, and perhaps most importantly, accurate central venous pressure (CVP) measurement was unavailable. CVP is crucial in this group of patients given its integral relationship to defining RV failure and impact of preload [35]. All of these parameters would be important to include in subsequent prospective study design.

## Conclusions

A short PAAT suggestive of raised PVR is found in a high proportion of patients with sepsis. A significant proportion of patients with sepsis have RV–PA uncoupling. Shorter PAAT and lower RV–PA coupling ratios seem to be associated with increasing severity of RVD; however, much remains unknown about their therapeutic and prognostic role above standard parameters. Categorising patients with sepsis-related RVD into those with and without raised PVR and RV–PA uncoupling could provide incremental information to help differentiate ‘RVD phenotypes’ and enable more precise definitions in future prospective study design.

## Supplementary Information

**Additional file 1.** Table of baseline characteristics of ICU survivors and nonsurvivors.

**Additional file 2.** Table and descriptive plots of echo parameters and P/F ratios.

**Additional file 3.** Table of echo and biochemical data in those who received mechanical ventilation.

**Additional file 4.** Table and descriptive plots of RV function and blood gas parameters on ICU admission.

## Data Availability

The data and material used in this article belong to the corresponding author and can be accessed with permission.

## Abbreviations

RVD: Right ventricular dysfunction
RVOT: Right ventricular outflow tract
PVR: Pulmonary vascular resistance
PAAT: Pulmonary artery acceleration time
PAATc: Pulmonary artery acceleration time corrected for heart rate
VTI: Velocity time integral
ET: Ejection time
RV–PA: Right ventricular–pulmonary artery
PASP: Pulmonary artery systolic pressure
TAPSE: Tricuspid annular planar systolic excursion
TRVmax: Tricuspid regurgitation maximum velocity
TTE: Transthoracic echocardiography
PWD: Pulsed wave Doppler
MSN: Midsystolic notching
