# Supplementary material for: Right ventricular outflow tract Doppler flow analysis and pulmonary arterial coupling by transthoracic echocardiography in sepsis: a retrospective exploratory study
Source: Crit Care. 2022 Oct 3;26:303. doi: 10.1186/s13054-022-04160-4 (PMC9527734; doi:10.1186/s13054-022-04160-4)
Supplement: Supplementary file 1 — Additional file 1. Table of baseline characteristics of ICU survivors and nonsurvivors. [file 13054_2022_4160_MOESM1_ESM.docx]

| **Patient characteristics** | **ICU Survivors (n=96)** | **ICU Non-Survivors (n=10)** | **P value** |
| --- | --- | --- | --- |
| Age | 65 ±13 | 65 ±15 | 0.89 |
| Male | 55 (57%) | 6 (60%) | 1 |
| Weight (Kg) | 94 ±35 | 79 ±33 | 0.25 |
| **APACHE 3** | **72 ± 23** | **101 ±26** | **<0.001** |
| ICU length of stay (days) | 4.1 (4) | 3.1 (4.6) | 0.80 |
| Time to TTE (days) | 2 (1.5) | 2 (0.75) | 0.24 |
| Invasive ventilation | 27 (27%) | 6 (60%) | 0.06 |
| **Renal replacement therapy** | **9 (9%)** | **4 (40%)** | **0.01** |
| Vasoactive therapy | 75 (78%) | 10 (100%) | 0.09 |
| **HR bpm during TTE** | **84 (23)** | **115 (11)** | **<0.001** |
| Atrial fibrillation during TTE | 11 (11%) | 1 (10%) | 0.89 |
| Chronic cardiovascular disease | 4 (4%) | 0 | 1 |
| Chronic respiratory disease | 17 (18%) | 2 (20%) | 1 |
| Chronic renal disease | 5 (5%) | 0 | 1 |
| Cirrhosis | 3 (3%) | 1 (10%) | 0.33 |
| **Immunosuppressed** | **13 (14%)** | **4 (40%)** | **0.05** |
| Lactate on admission, mmol/L | 1.6 (2.4) (n=85) | 2.3 (2.5) (n=9) | 0.09 |
| pH on admission | 7.36 ± 0.09 (n=83) | 7.33 ±0.09 (n=9) | 0.34 |
| P/F ratio on admission | 235 (175) (n=83) | 227 (221) (n=9) | 0.99 |
| PaC02 on admission | 38 (11) (n=83) | 41 (17) (n=9) | 0.56 |
| Bilirubin | 11 (15) (n=76) | 17 (20) (n=9) | 0.26 |
| Creatinine | 134 (156) (n= 94) | 122 (134) (n= 10) | 0.63 |

Supplemental Table 1 – baseline characteristics between survivors and non survivors. Data presented as absolute value and (%), mean ± standard deviation or median (interquartile range).
