# Supplementary material for: Right ventricular outflow tract Doppler flow analysis and pulmonary arterial coupling by transthoracic echocardiography in sepsis: a retrospective exploratory study
Source: Crit Care. 2022 Oct 3;26:303. doi: 10.1186/s13054-022-04160-4 (PMC9527734; doi:10.1186/s13054-022-04160-4)
Supplement: Supplementary file 2 — Additional file 2. Table and descriptive plots of echo parameters and P/F ratios. [file 13054_2022_4160_MOESM2_ESM.docx]

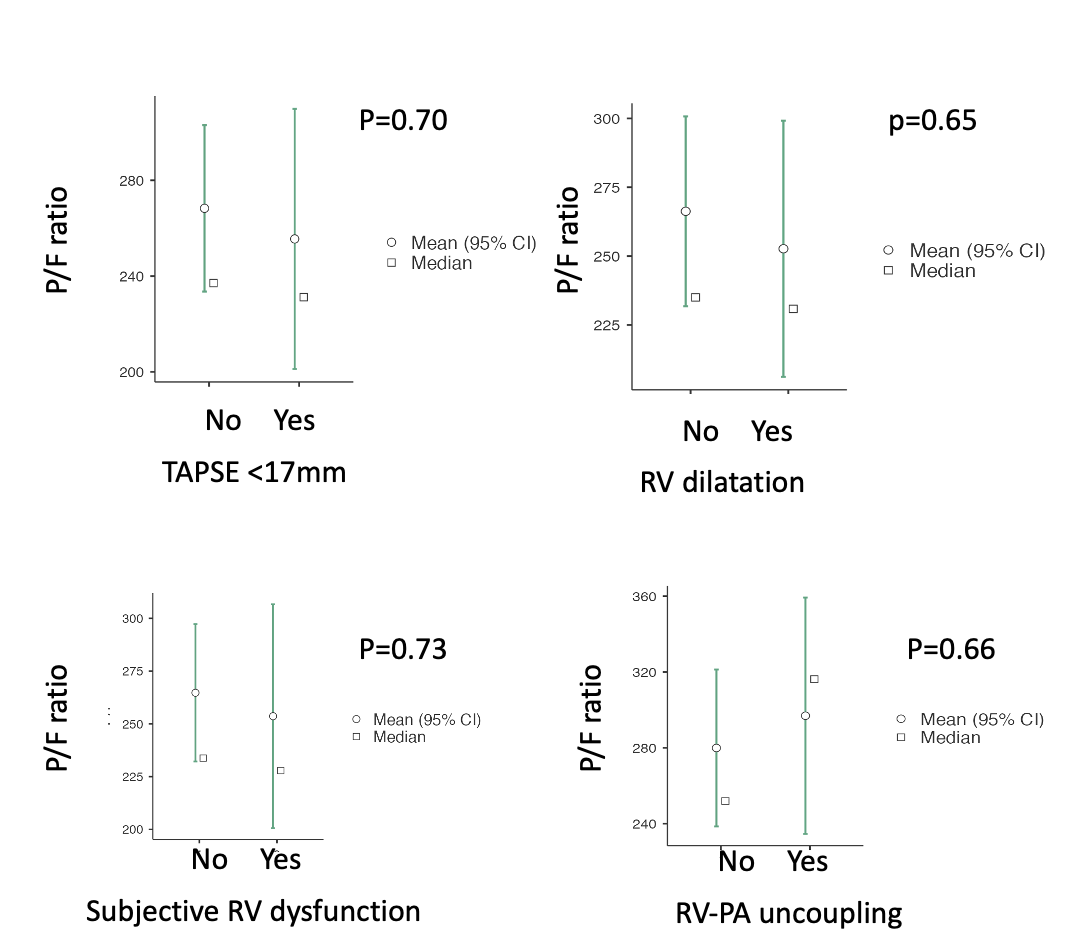


Supplementary material 2– No significant differences in admission P/F ratio in those with and without RVD defined by subjective, dilation, TAPSE and RV-PA uncoupling parameters (TAPSE/PASP ratio <0.31mm/mmHg= RV-PA uncoupling).

|  | P/F $\leq$ 100 (n=6) | P/F 101-199 (n=32) | P/F 200-299 (n=20) | P/F $\geq$300(n=34) | P value |
| --- | --- | --- | --- | --- | --- |
| PAAT (msec) | 90 $\pm$ 19 | 85$\pm$16 | 94$\pm$21 | 94$\pm$22 | 0.25 |
| **RVOT VTI (cm)** | **13** $\boldsymbol{\pm}$**5.4** | **12.2** $\boldsymbol{\pm}$ **3.7** | **13.9**$\boldsymbol{\pm}$ **5** | **15**$\boldsymbol{\pm3.6}$ | **0.05*** |
| **RVOT ET (ms)** | **247**$\boldsymbol{\pm}$**59** | **244**$\boldsymbol{\pm}$**50** | **291**$\boldsymbol{\pm}$**42** | **291** $\boldsymbol{\pm}$**55** | **0.004**** |
| HR | 94 $\pm$ 25 | 94 $\pm$19 | 88$\pm$18 | 81 $\pm$21 | 0.12 |

Supplementary material 2 - RVOT measurements and P/F ratio severity categories.

*Post hoc TukeyHSD revealed significant difference between P/F $\geq$300 and P/F 101-109 groups only.

** Post hoc TukeyHSD revealed significant difference between P/F $\geq$300 and P/F 101-109 and P/F 101-109 and P/F 200-299 groups only. HR= heart rate. Data presented as mean ± standard deviation.
