# Supplementary material for: Right ventricular outflow tract Doppler flow analysis and pulmonary arterial coupling by transthoracic echocardiography in sepsis: a retrospective exploratory study
Source: Crit Care. 2022 Oct 3;26:303. doi: 10.1186/s13054-022-04160-4 (PMC9527734; doi:10.1186/s13054-022-04160-4)
Supplement: Supplementary file 3 — Additional file 3. Table of echo and biochemical data in those who received mechanical ventilation. [file 13054_2022_4160_MOESM3_ESM.docx]

|  | No mechanical ventilation | Mechanical ventilation | P value |
| --- | --- | --- | --- |
| **Admission P/F ratio** | **300 ± 132** | **186 ±106** | **<0.001** |
| Admission PaC02 | 37 (10) | 39 (12) | 0.78 |
| **Admission lactate** | **1.5 (1.7)** | **2.8 (4.3)** | **0.002** |
| PAAT (msec) | 90 ± 21 | 91 ± 19 | 0.83 |
| PAATc (msec) | 80 (42) | 73 (31) | 0.25 |
| RVOT VTI (cm) | 14.3± 4.3 | 12.7 ± 3.5 | 0.07 |
| **RVOT ET (ms)** | **281 (75)** | **258 (67)** | **0.004** |
| TRV (m/s) | 2.78± 0.52 | 2.71 ± 0.62 | 0.66 |
| TAPSE (mm) | 20 ± 6 | 19 ± 4 | 0.40 |
| TAPSE/PASP ratio | 0.49 (0.26) | 0.47 (0.18) | 0.66 |

Supplementary material 3 – Biochemical and echocardiographic differences in those who received mechanical ventilation in ICU. Significantly higher lactate, lower P/F ratios and RVOT ET (ms) in those who received mechanical ventilation. Data presented as mean ± standard deviation or median (interquartile range).
