# Supplementary material for: Right ventricular outflow tract Doppler flow analysis and pulmonary arterial coupling by transthoracic echocardiography in sepsis: a retrospective exploratory study
Source: Crit Care. 2022 Oct 3;26:303. doi: 10.1186/s13054-022-04160-4 (PMC9527734; doi:10.1186/s13054-022-04160-4)
Supplement: Supplementary file 4 — Additional file 4. Table and descriptive plots of RV function and blood gas parameters on ICU admission. [file 13054_2022_4160_MOESM4_ESM.docx]

|  | TAPSE | | P value |
| --- | --- | --- | --- |
|  | <17mm | $\geq$17mm |  |
| P/F ratio | 256 ± 130 | 268 ± 142 | 0.71 |
| PaC02 (mmHg) | 44.1 ± 14.3 | 38.5 ± 11.6 | 0.07 |
| **pH** | **7.32** ± **0.07** | **7.36** ± **0.09** | **0.04** |
| Lactate (mmol/l) | 1.9 (2.0) | 1.6 (2.3) | 0.68 |

Supplementary material 4 – Admission arterial blood gas results in those with RVD defined by TAPSE < 17mm. Those with TAPSE <17mm had a significantly lower admission pH. There was a trend towards a higher C02 in those with TAPSE <17mm that was not statistically significant. Data presented as mean ± standard deviation or median (interquartile range).


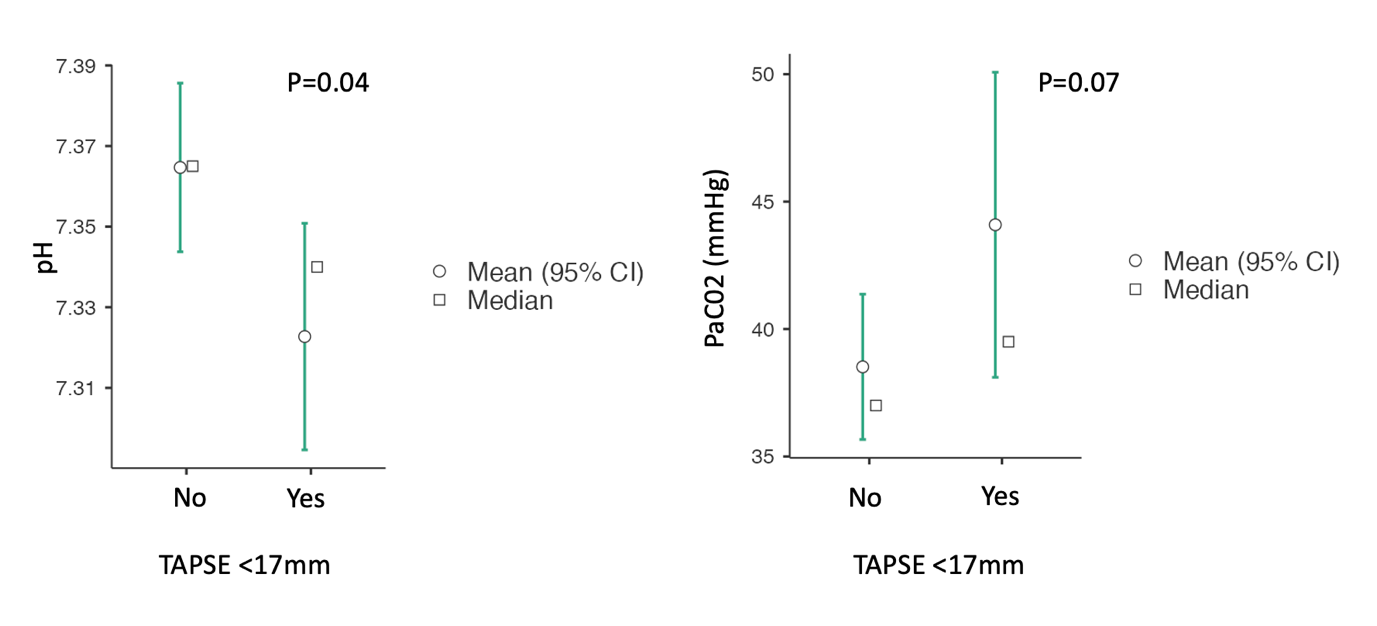

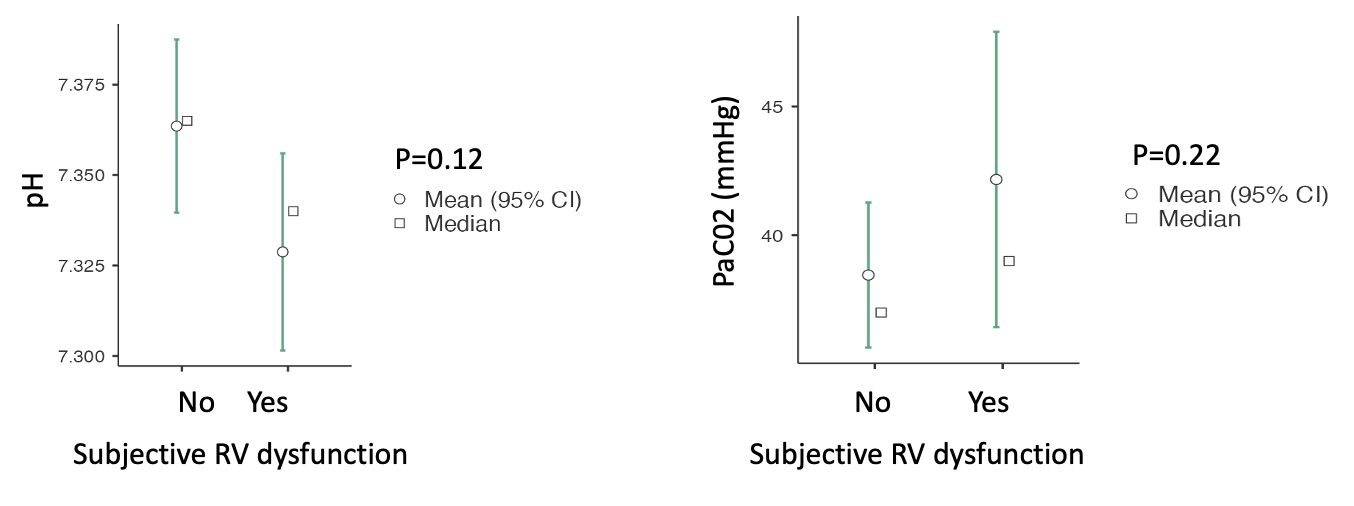


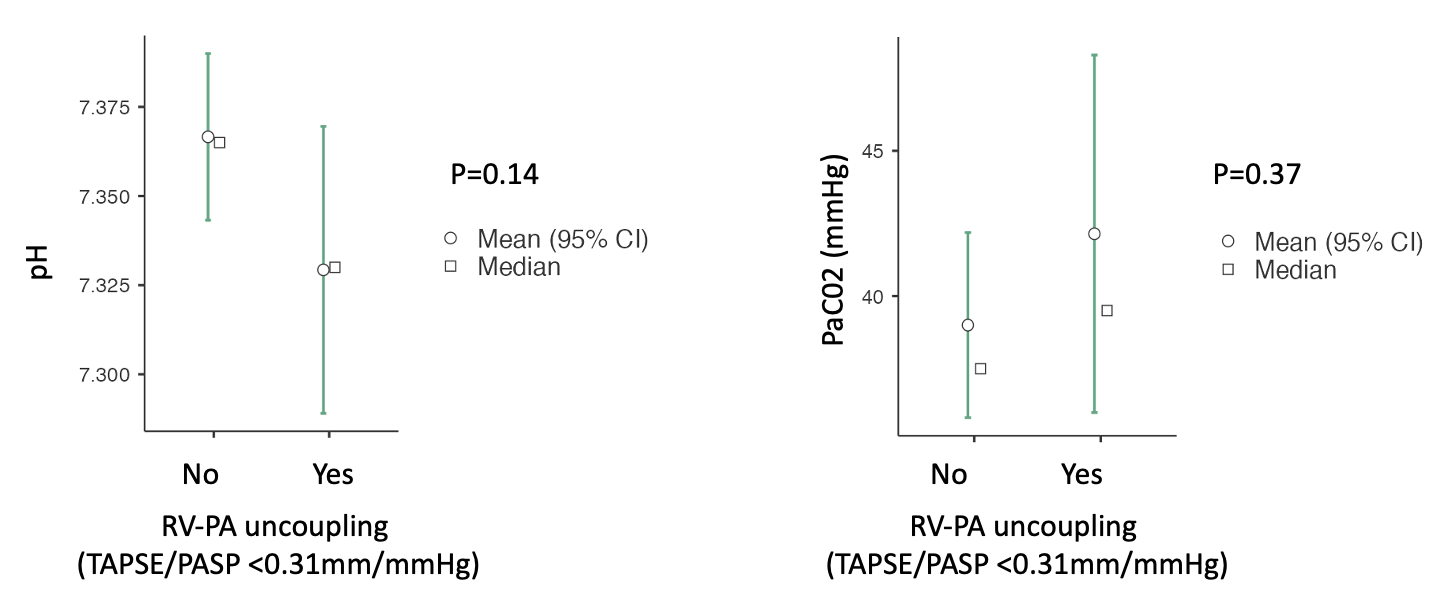

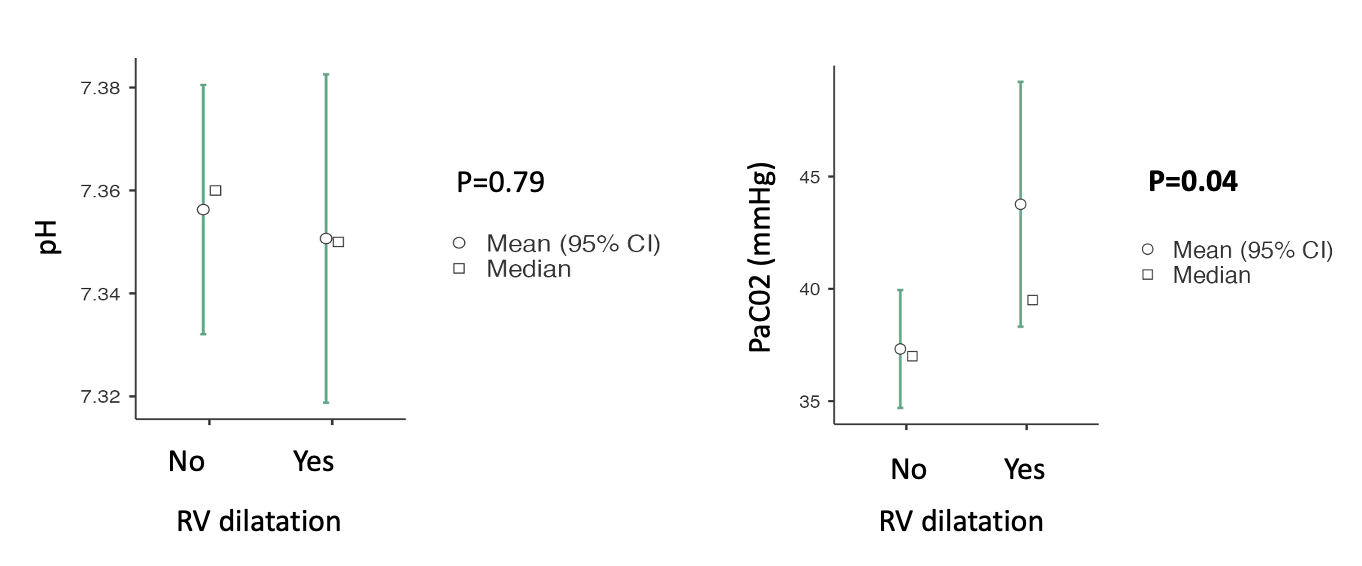


Supplementary material 4 – Bar plots of RVD by TAPSE, subjective, RV-PA uncoupling and RV dilation against admission pH and PaC02. Those with reduced TAPSE had significantly lower admission pH 7.32 ± 0.07 vs. 7.36 ± 0.09, p=0.04. Those with RV dilation had a higher PaC02 on admission (39.5mmHg (11.8) vs. 37mmHg (18.8), p=0.04.
